# First DNA metabarcoding diet assessment on the critically endangered Tricolour Langur, *Presbytischrysomelascruciger*

**DOI:** 10.3897/BDJ.12.e124990

**Published:** 2024-07-08

**Authors:** Roberta Chaya Tawie Tingga, Jack Liam, Bilhan Deli, Mohd Luqman Anuar, Ahmad Ampeng, Badrul Munir Md-Zain

**Affiliations:** 1 Department of Biological Sciences and Biotechnology, Faculty of Science and Technology, Universiti Kebangsaan Malaysia, 43600, Bangi, Selangor, Malaysia Department of Biological Sciences and Biotechnology, Faculty of Science and Technology, Universiti Kebangsaan Malaysia 43600, Bangi, Selangor Malaysia; 2 Centre for Pre-University Studies, Universiti Malaysia Sarawak, 94300, Kota Samarahan, Sarawak, Malaysia Centre for Pre-University Studies, Universiti Malaysia Sarawak, 94300 Kota Samarahan, Sarawak Malaysia; 3 Forest Department Sarawak, Bangunan Baitul Makmur II, Medan Raya, 93050, Petra Jaya, Kuching, Sarawak, Malaysia Forest Department Sarawak, Bangunan Baitul Makmur II, Medan Raya, 93050 Petra Jaya, Kuching, Sarawak Malaysia

**Keywords:** Malaysian Borneo, primate, diet, conservation, metabarcoding, critically endangered subspecies

## Abstract

*Presbytischrysomelascruciger* or also known as the Tricolour langur—is rare, endemic to Sarawak and Kalimantan in Borneo and classified as a critically endangered subspecies. The current *P.c.cruciger* population size is uncertain because the numbers are continuously decreasing. At present, there is no comprehensive scientific report on *P.c.cruciger* in Sarawak, although this subspecies is known to inhabit Maludam area. Recent first sighting of *P.c.cruciger* in Jemoreng Sarawak presents a research opportunity to study its feeding from a molecular ecology perspective. Herein, we report the first findings on the dietary intake of *P.c.cruciger* using a high-throughput DNA metabarcoding approach. We emphasise the diet intake of *P.c.cruciger* from Jemoreng Protected Forest in Sarawak using DNA metabarcoding of the trnL region. Preliminary findings revealed 11 amplicon sequence variants (ASV) classified into one phylum, four classes, four orders, four families, three genera and three plant species. *Fibraureatinctoria* (akar kuning; Family Menispermaceae), *Poikilospermumsuaveolens* (akar jangkang; Family Urticaceae) and *Litchichinensis* (lychee; Family Sapindaceae) were the three main plant species that were consumed by *P.c.cruciger*. Understanding the dietary intake of *P.c.cruciger* is of paramount importance for their conservation and management of the habitat areas where their population resides.

## Introduction

The genus *Presbytis* is a diverse primate from the Old World Monkey (Meyer et al. 2011), with the largest number of species amongst the subfamily Colobinae (Family: Cercopithecidae) (Roos et al. 2014). There are five species of *Presbytis* recorded in Sarawak and *Presbytischrysomelas* is a unique species endemic to Borneo (Md-Zain et al. 2022, Noor-Faezah et al. 2023, Nur-Aizatul et al. 2024). There are two subspecies of *P.chrysomelas* — *P.c.cruciger* and *P.c.chrysomelas* (Roos et al. 2014); both of these subspecies have a distinct colour morphology (Groves 2001, Ampeng et al. 2024); however, historical records of mixed-troops of both subspecies suggest that their variation should be considered as colour variants rather than different subspecies (Phillipps and Phillipps 2018). Unlike *P.c.chrysomelas* with two colour variations (black and white) (Ampeng 2003), *P.c.cruciger* has three colour variations: head, shoulders, sides of the abdomen, thigh and calves have red–orange hair; hair on its cheeks, under its chest and abdomen are white; and arms, hands, feet and lines on the back are black (Rifqi et al. 2019, Ampeng et al. 2024).

*P.c.cruciger* is endemic to Sarawak and Kalimantan, along with its sister taxon, *P.c.chrysomelas* and has been sighted in Maludam (Sarawak) and Danau Sentarum (Kalimantan) (Phillipps and Phillipps 2018, Rifqi et al. 2019, Santoso et al. 2023a, Santoso et al. 2023b). Although its distribution is confined to Maludam, no comprehensive scientific information is available. Recently, Ampeng et al. (2024) made an important discovery about the first sighting of *P.c.cruciger* in Jemoreng Protected Forest, Sarawak. This is the first scientific discovery to describe the presence of *P.c.cruciger* in Sarawak in detail. According to Nijman et al. (2020), it is a rare primate, accounting for < 5% of the primate historical distribution. Based on the International Union for Conservation of Nature’s Red ist category, *P.c.cruciger* has been classified as a critically endangered subspecies (IUCN 2024). Its population size has decreased by ~ 80% over the past 30 years (Nijman et al. 2020). The increasing land conversion into oil palm plantations has become a major threat to the survival of *P.c.cruciger* (Nijman et al. 2020). *P.c.cruciger* is one of the most neglected transboundary primates in Sarawak and Kalimantan (Md-Zain 2019, Ampeng et al. 2024).

Considering the possible extinction of this subspecies, it is essential to take immediate action and adopt relevant measures to protect its survival. Furthermore, there is a lack of research regarding this subspecies, such as its feeding ecology and habitat utilisation (Santoso et al. 2023b, Ampeng et al. 2024). There are insufficient data to fully comprehend this species’ ecology in its native environment (Nijman et al. 2020). A thorough review of how this species is affected by its ecological communities, habitat change and revising conservation plans requires a prior understanding of their diets in the wild (Osman et al. 2020, Abdullah-Fauzi et al. 2022, Mohd-Radzi et al. 2022, Osman et al. 2022). Primate diets of *P.c.cruciger* have been described from the Kalimantan population (Santoso et al. 2023a, Santoso et al. 2023b) and comprehensive feeding ecology information in Sarawak is available for its sister taxon, *P.c.chrysomelas*, in Samunsam Wildlife Sanctuary (Ampeng 2007, Ampeng and Md-Zain 2007, Ampeng and Md-Zain 2012). At present, dietary studies of primates can be conducted using a DNA metabarcoding approach as an alternative to direct ecological observation. This next-generation sequencing (NGS) technique that utilises a metabarcoding diet approach is suitable for studying the diet of the rarely sighted *P.c.cruciger*. An advantage of the DNA metabarcoding approach is that it identifies multiple species from a single DNA marker using degraded samples, such as faeces (Khairulmunir et al. 2023, Gani et al. 2024a, Gani et al. 2024b). As it is rare to encounter the endangered *P.c.cruciger* in the wild, obtaining faecal samples to evaluate its diet is necessary and beneficial.

## Materials and Methods

**Figure 1.**
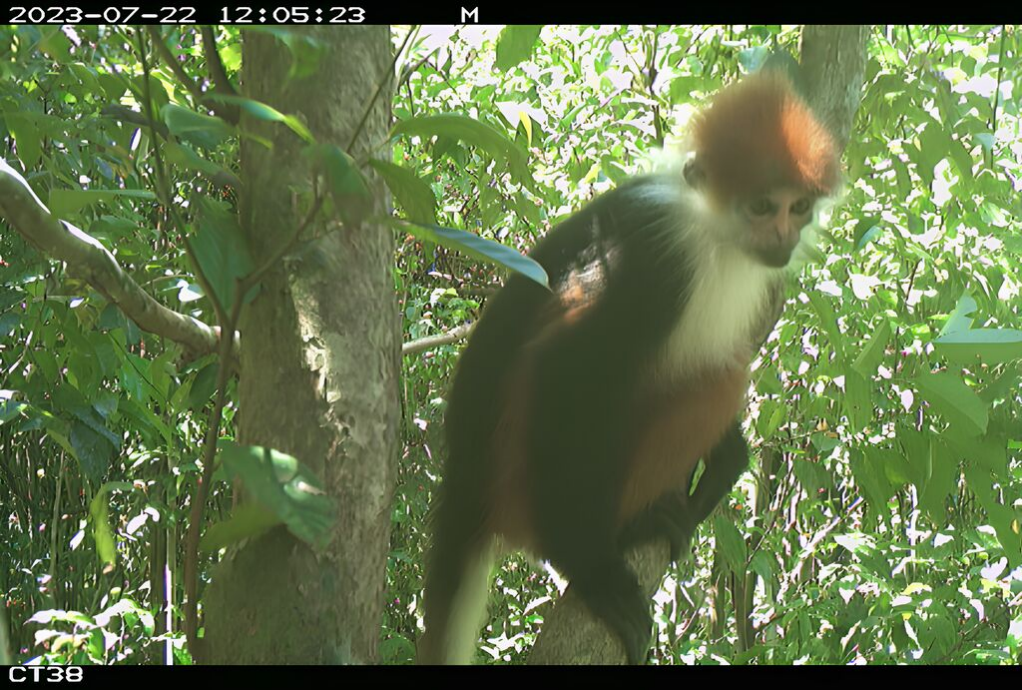
Tricolour Langur of Jemoreng Protected Forest captured using a camera-trap method.

The first sighting of *P.c.cruciger* in Jemoreng Protected Forest (JPF), Sarawak (2°42′00′′N, 111°39′00′′E) was made during scientific surveys conducted by the Forest Department Sarawak using a camera-trap method (Fig. 1) (Ampeng et al. 2024). During this survey, only a single faecal sample of *P.c.cruciger* was successfully obtained from the study site. Total genomic DNA was extracted using the QIAamp PowerFecal Pro DNA Kit according to the manufacturer’s protocol. The extraction produced high-quality DNA and its concentration was quantified using a Hercuvan Nucleic Acid Analyzer. Genetic identification of the faecal sample was performed using D-*loop* region sequences (Meyer et al. 2011) (Table 1) and species confirmation was performed via GenBank BLASTN (pairwise distance: 95.1% similarity with *P.chrysomelas*). Genomic DNA product of *P.c.cruciger* was sent to Apical Scientific Sdn Bhd for further NGS processing. Amplicon sequencing of the diet was performed using locus-specific primers of the trnL gene (P6 loop region) with overhang adapters (Taberlet et al. 2007) (Table 1). To accomplish library amplification, KOD-Multi & Epi-® (Toyobo) was utilised. Dual indices were affixed to the amplicon PCR using the Illumina Nextera XT Index Kit version 2, in compliance with the manufacturer’s standard protocol. The quality of the libraries was assessed using an Agilent Bioanalyzer 2100 System with an Agilent DNA 1000 Kit and via fluorometric quantification using Helixyte Greenä Quantifying Reagent. The library was normalised and pooled following the protocol recommended by Illumina and the MiSeq platform was used to perform a 150-paired end sequencing. The raw FASTQ data were filtered and assessed using fastqc (https://www.bioinformatics.babraham.ac.uk/projects/fastqc/). R Studio version 2023.09.1 with DADA2 packages analysed the obtained NGS data. All amplicon sequence variants (ASV) data were generated by filtering, denoising, merging and deleting chimeras using DADA2 (Callahan et al. 2016). The obtained ASV data were imported and used to perform dietary characterisation analysis in R Studio.

## Results and Discussion

**Figure 2.**
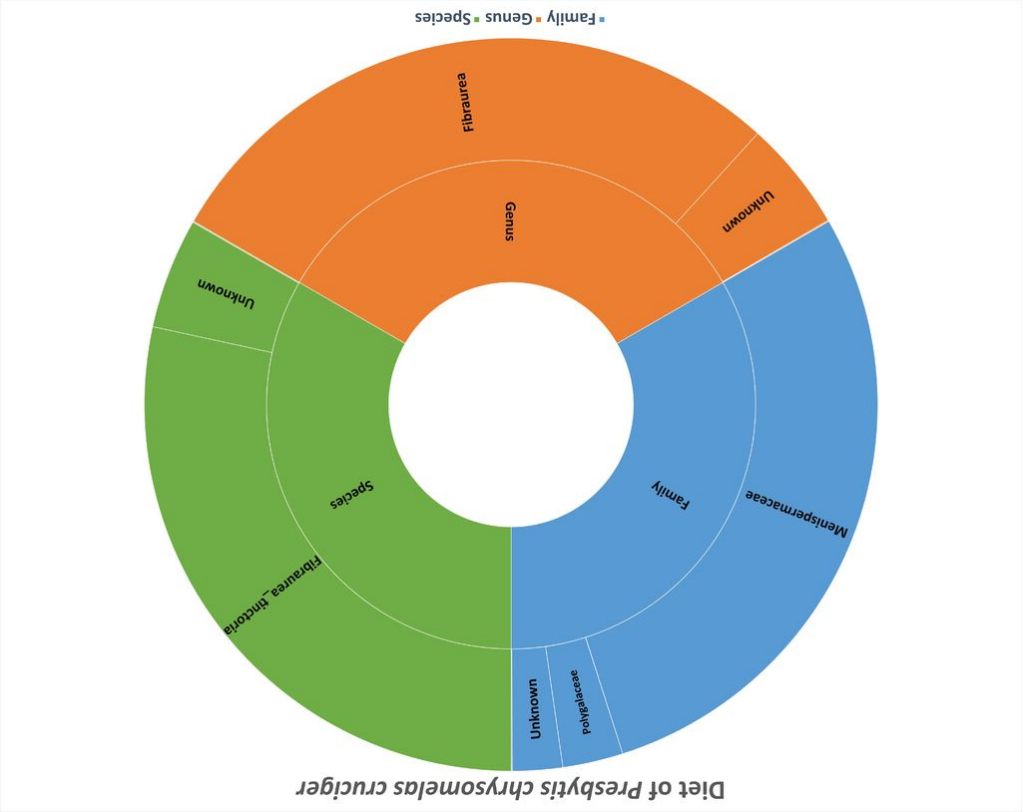
Sunburst taxonomy on the diet of *P.c.cruciger* from the Jemoreng Protected Forest.

Amplicon sequencing of the trnL gene from a single individual of *P.c.cruciger* successfully generated 87,473 raw reads, followed by filtered (85,116), denoised (85,095) and merged (83,816) data reads. The final non-chimeric sequences consisted of 82,488 reads, which resulted in 11 ASVs for the diet profiling analyses. From the total non-chimeric reads acquired, 6.62% of the sequences were not assigned to any taxonomy classification. From the 11 acquired ASVs, one phylum, four classes, four orders, four families, three genera and three species were identified. Streptophyta is the only identified phylum in the *P.c.cruciger* faecal sample. Fig. 2 shows the sunburst chart of the taxonomic composition and the relative abundance of the *P.c.cruciger* faecal sample at the family, genus and species levels. Menispermaceae (85.22%) is the most abundant at the family level, followed by Polygalaceae (8.01%), Urticaceae (0.12%) and Sapindaceae (0.03%). Meanwhile, the most abundant at the genus and species level, with 85.22%, are *Fibraurea* and *Fibraureatinctoria*. Other identified genera included *Poikilospermum* (0.12%) and *Litchi* (0.03%), while other identified species were *Poikilospermumsuaveolens* (0.12%) and *Litchichinensis* (0.03%).

Overall, there were three plant species which were successfully identified in their diet, based on the NGS approach: *F.tinctoria* (akar kuning), *P.suaveolens* (akar jangkang) and *L.chinensis* (lychee). Meanwhile, 14.63% were not identified at the species level. *F.tinctoria*, from the family Menispermaceae, is typically found in lowland forests — either primary, secondary or disturbed — at altitudes up to 1,200 m a.s.l. (MyBIS 2024). In Sarawak, this plant species is frequently found in peat swamp forests, including JPF, where the faecal sample of *P.c.cruciger* was collected. In addition, *F.tinctoria* was discovered in the diet of the endemic Bornean Maroon langur, *Presbytisrubicunda* (Ehlers-Smith et al. 2013) at Sabangau, Kalimantan. However, in contrast to *P.c.cruciger*, *F.tinctoria* was not the main preference for *P.rubicunda* and only 1.6% of their mean monthly feeding time was utilised to consume this plant. Meanwhile, *P.suaveolens* was discovered to be a part of the diet of another *Presbytis* species, *P.fredericae*, although in low abundance (Suryana 2010), indicating that *F.tinctoria* is a common plant species consumed by the genus *Presbytis*.

Reportedly, *P.c.cruciger* of Danau Sentarum National Park (West Kalimantan) feeds on 27 types of plant species (Santoso et al. 2023b). Gita susu (*Willughbeiacoriacea*), Merepat (unidentified) and Karet (*Heveabrasiliensis*) were their main diets. Concerning feeding composition, Santoso et al. (2023b) described that *P.c.cruciger* mainly preferred leaves (50%), followed by fruits (30%) and seeds (20%). Furthermore, *P.c.cruciger* preferred feeding from trees (89%) and lianas (11%) (Santoso et al. 2023b). Plant species *F.tinctoria*, *P.suaveolens* and *L.chinensis* have small fruits. Previous studies have also reported that *P.c.cruciger* usually consumes small- to medium-sized fruits with a diameter of 0.5–5 cm (Santoso et al. 2023b). In another study, 10 plant species were identified as the primary food source of *P.c.cruciger*, including *Grewiapaniculata*, *Diospyros* sp. and *Pternandragaleata* (Santoso et al. 2023a). However, its sister taxon, *P.c.chrysomelas*, from Samunsam Wildlife Sanctuary (Western Sarawak), consumes Dipterocarpaceae, followed by Anacardiaceae and Myrtaceae (Ampeng 2007). When there are several diet options, the animal’s preference for a specific food is determined by its palatability (‌Prescott 2006, Kirschner et al. 2014). A few factors may contribute to the palatability for a primate, such as food availability, food abundance and distribution, nutritional content and energy (Solanki et al. 2008, Grueter et al. 2009, Osman et al. 2020, Strier 2021).

The preliminary dietary intake data of the rare *P.c.cruciger* is important for their conservation strategies, particularly for plant species that are highly consumed by this primate. Their population has been decreasing for the past few years, with an unknown population size at present. Maludam National Park was the only known record for the existing distribution of *P.c.cruciger* in Sarawak (Nijman et al. 2020). Thus, the recent discovery of *P.c.cruciger* in JPF has provided research opportunities for comprehensive feeding ecological studies. Knowing the importance of certain food resources, such as how the available food suits the nutritional demands, primates’ flexible diets, value of fallback foods and nutritional needs of the various species, will be essential for conservation strategy planning (Chapman et al. 2012).

In conclusion, these preliminary findings identified three plant species consumed by *P.c.cruciger*: *F.tinctoria* (Menispermaceae), *P.suaveolens* (Urticaceae) and *L.chinensis* (Sapindaceae). Acquiring knowledge and understanding their diets is essential for the conservation management purposes of *P.c.cruciger* and their preferred consumed plant where its population inhabit. Further research studies on its behavioural ecology and genetic aspects are necessary to comprehend the rare and critically endangered *P.c.cruciger* in Sarawak.

## Figures and Tables

**Table 1.** Primer sequences for the mtDNA D-*loop* region and chloroplast trnL gene/.

Primer	Sequence
hf_dloop_F	5′-GCCCTTATGTAATTCGTGCATTAC-3′
HV-1_r	5′-TGATAGACCCGTGATCCATC-3′
gtnrl	5′-TCGTCGGCAGCGTCAGATGTGTATAAGAGACAGGGGCAATCCTGAGCCAA-3′
htrnl	5′-GTCTCGTGGGCTCGGAGATGTGTATAAGAGACAGCCATTGAGTCTCTGCACCTATC-3′

## References

[B11348620] Abdullah-Fauzi N. A. F., Karuppannan K. V., Mohd-Radzi N. H. S., Gani M., Mohd-Ridwan A. R, Othman N., Haris H., Sariyati N. H., Aifat N. R., Abdul-Latiff M. A. B., Abdul-Razak M. F. A., Md-Zain B. M. (2022). Determining the dietary preferences of wild asian elephants (*Elephasmaximus*) in Taman Negara National Park, Malaysia based on sex and age using trnL DNA metabarcoding analysis. Zoological Studies.

[B11348654] Ampeng A. (2003). Densiti dan kepelbagaian primat diurnal di Taman Negara Tanjung Datu, Sematan, Sarawak.

[B11348662] Ampeng A. (2007). Ekologi dan kelakuan lotong penatat, *Presbytismelalophoschrysomelas* di Sanktuari Hidupan Liar Samunsam, Sarawak.

[B11348670] Ampeng A., Md-Zain B. M. (2007). A short note on methodology of detecting leaf monkeys (*Presbytismelalophoschrysomelas* and *Trachypithecuscristatus*) in Samunsam Wildlife Sanctuary, Sarawak. The Journal of Wildlife and Parks.

[B11348688] Ampeng A., Md-Zain B. M. (2012). Ranging patterns of critically endangered colobine, *Presbytischrysomelaschrysomelas*. The Scientific World Journal.

[B11348738] Ampeng A., Mohammad H., Liam J., George-Pau M., Osman S., Jinggong E. R., Borhan R., Hassim I., Abdul-Rahman M. F., Md-Nor S., Traeholt C., Md-Zain B. M. (2024). First recorded sighting of the critically endangered tricolour langur, *Presbytischrysomelascruciger* (Thomas, 1892) (Primates, Cercopithecidae), in Jemoreng Protected Forest, Sarawak, Malaysia. Check List.

[B11348779] Callahan B. J., McMurdie P. J., Rosen M. J., Han A. W., Johnson A. J. A., Holmes S. P. (2016). DADA2: High-resolution sample inference from Illumina amplicon data. Nature Methods.

[B11348801] Chapman C. A., Rothman J. M., Lambert J. E., Mitani J., Call J., Kappeler P., Palombit R., Silk J. (2012). The evolution of primate societies.

[B11348837] Ehlers-Smith D. A., Husson S. J., Ehlers Smith Y. C., Harrison M. E. (2013). Feeding ecology of red langurs in Sabangau Tropical Peat-Swamp Forest, Indonesian Borneo: Extreme granivory in a non-masting forest. American Journal of Primatology.

[B11348850] Gani M., Mohd-Ridwan A. R., Sitam F. T., Zubaidah Kamarudin Z., Selamat S. S., Mohd N., Karuppannan K. V., Md-Zain B. M. (2024). Habitat shapes the gut microbiome diversity of Malayan tigers (*Pantheratigrisjacksoni*) as revealed through metabarcoding 16S rRNA profiling. World Journal of Microbiology & Biotechnology.

[B11772962] Gani Millawati, Sitam Frankie Thomas, Kamarudin Zubaidah, Selamat Siti Suzana, Awang Nik Mohd Zamani, Muhd-Sahimi Hani Nabilia, Wong Michael, Selat Baharim, Abdullah-Halim Nur Fatin Khairunnisa, Yong Lim Shu, Yoke Ling Fong, Yaakop Salmah, Mohd-Ridwan Abd Rahman, Md-Zain Badrul Munir (2024). Unveiling prey preferences of endangered wild Malayan tiger, *Pantheratigrisjacksoni*, in Peninsular Malaysia through scat analysis via COI DNA metabarcoding. Nature Conservation.

[B11348934] Groves C, P. (2001). Primate Taxonomy.

[B11348942] Grueter C. C., Li D., Ren D., Vanschaik C. P. (2009). Dietary profile of *Rhinopithecusbieti* and its socio ecological implications. International Journal of Primatology.

[B11348968] IUCN The IUCN Red List of Threatened Species. IUCN Red List of Threatened Species; IUCN. https://www.iucnredlist.org/.

[B11349028] Khairulmunir M., Gani M., Karuppannan K. V., Mohd-Ridwan A. R., Md-Zain B. M. (2023). High-throughput DNA metabarcoding for determining the gut microbiome of captive critically endangered Malayan tiger (*Pantheratigrisjacksoni*) during fasting. Biodiversity Data Journal.

[B11349064] Kirschner A., Putnam L., Calvin A., Irlbeck N. Browse species preference and palatability of *Colobus guereza kikuyuensis* at the Denver Zoological Gardens. https://nagonline.net/wp-content/uploads/2014/02/6_KIRSCHNE.pdf.

[B11368425] Md-Zain B. M. (2019). Transboundary primates: Current status and conservation genetics.

[B11349082] Md-Zain B. M., Abdul-Latiff M. A. B., Mohd-Ridwan A. R., Najmuddin M. F. (2022). Primat Semenanjung Malaysia.

[B11349120] Meyer D., Rinaldi Ir. D., Ramlee H., Perwitasari-Farajallah D., Hodges J. K., Roos C. (2011). Mitochondrial phylogeny of leaf monkeys (genus *Presbytis*, Eschscholtz, 1821) with implications for taxonomy and conservation. Molecular Phylogenetics and Evolution.

[B11349197] Mohd-Radzi N. H. S., Karuppannan K. V., Abdullah-Fauzi N. A. F., Mohd-Ridwan A. R., Othman N., Abdul-Latiff M. A. B., Gani M., Abdul-Razak M. F. A., Md-Zain B. M. (2022). Determining the diet of wild Asian elephants (*Elephasmaximus*) at human–elephant conflict areas in Peninsular Malaysia using DNA metabarcoding. Biodiversity Data Journal.

[B11349222] MyBIS Malaysia Biodiversity Information System. Ministry of natural resources and environmental sustainability, Malaysia Biodiversity Centre & Forest Research Institute Malaysia. https://www.mybis.gov.my/.

[B11349242] Nijman V., Cheyne S., Traeholt C., Setiawan A. (2020). Presbytis chrysomelas ssp. chrysomelas. The IUCN Red List of Threatened Species 2020: e.T136857A17987458.

[B11349251] Noor-Faezah M., Nur-Aizatul T., Tingga R. C. T., Mohamad-Fhaizal B., Mohd-Azlan J., Azroie D., Md-Zain B. M., Abdul-Latiff M. A. B., Mohd-Ridwan A. R. (2023). A brief review of Bornean banded langur *Presbytischrysomelas* (Müller, 1838) of Sarawak. Journal of Wildlife and Biodiversity.

[B11772994] Nur-Aizatul Tukiman, Mohd-Ridwan Abd Rahman, Noor-Faezah Mohammad, Tingga Roberta Chaya Tawie, Bukhori Mohamad Fhaizal, Mohd-Azlan Jayasilan, Denel Azroie, Abdul-Latiff Muhammad Abu Bakar, Md-Zain Badrul Munir (2024). Preliminary assessment of group composition and activity pattern of the critically endangered Bornean Banded Langur *Presbytischrysomelaschrysomelas* in Tanjung Datu National Park. Biodiversity Data Journal.

[B11349268] Osman N. A., Abdul-Latiff M. A. B., Mohd-Ridwan A. R., Yaakop S., Nor S. M., Md-Zain B. M. (2020). Diet composition of the wild stump-tailed macaque (*Macacaarctoides*) in Perlis State Park, Peninsular Malaysia, using a chloroplast trnL DNA metabarcoding approach: A preliminary study. Animals.

[B11349434] Osman N. A., Abdul-Latiff M. A. B., Mohd-Ridwan A. R., Yaakop S., Karuppannan K. V., Md-Zain B. M. (2022). Metabarcoding data analysis revealed the plant dietary variation of longtailed macaque *Macacafascicularis* (Cercopithecidae, Cercopithecinae) living in disturbed habitats in Peninsular Malaysia. Biodiversity Data Journal.

[B11349445] Phillipps Q., Phillipps K. (2018). Phillipps’ field guide to the mammals of Borneo and their ecology: Sabah, Sarawak, Brunei, and Kalimantan.

[B11349471] ‌Prescott M. (2006). Primate sensory capabilities and communication signals: implications for care and use in the laboratory. Primate senses and communication 2006..

[B11349186] Rifqi M. A., Pambudi T., Khotiem M., Gesshaa A. A. (2019). Bornean banded Langur *Presbytischrysomelascruciger* in Danau Sentarum National Park, West Kalimantan, Indonesia. SEAVR.

[B11349482] Roos C., Boonratana R., Supriatna J., Fellowes J. R., Groves C. P., Nash S. D., Ryland A. B., Mittermeier R. A. (2014). An updated taxonomy and conservation status review of Asian primates. Asian Primates Journal.

[B11349529] Santoso N., Diva A. M., Nurul-Fauziah N., Sutopo (2023). Cohabitation study of tricolour langur (Presbytischrysomelasssp.cruciger) and proboscis monkey (*Nasalislarvatus*) in Bukit Semujan Danau Sentarum National Park. Jurnal Ilmu Kehutanan.

[B11349510] Santoso N., Sutopo, Meo L. E., Nurul-Fauziah N., Diva A. M. (2023). Preliminary study: feeding ecology and daily activity of three colored langur (Presbytischrysomelassspcruciger Thomas, 1892) In Danau Sentarum National Park.. Biotropia: The Southeast Asian Journal of Tropical Biology.

[B11349548] Solanki G. S., Kumar A., Sharma B. K. (2008). Feeding ecology of *Trachypithecuspileatus* in India.. International Journal of Primatology.

[B11349585] Strier K. B. (2021). Primate behavioral ecology (6th ed.)..

[B11349602] Suryana D. (2010). Study of feeding behaviour and palatability in Javan fuscous langurs (*Presbytisfredericae* Sody, 1930) at forest area and rubber plantation Gutomo Village, Pekalongan District, Province of Central Java.

[B11349610] Taberlet P., Coissac E., Pompanon F., Gielly L., Miquel C., Valentini A., Vermat T., Corthier G., Brochmann C., Willerslev E. (2007). Power and limitations of the chloroplast trnL (UAA) intron for plant DNA barcoding. Nucleic Acids Research.

